# Regulation of plant responses to biotic and abiotic stress by receptor-like cytoplasmic kinases

**DOI:** 10.1007/s44154-022-00045-2

**Published:** 2022-06-29

**Authors:** Xiangxiu Liang, Jie Zhang

**Affiliations:** 1 College of Life Sciences, South China Agricultural University, Guangzhou, 510642 China; 2 State Key Laboratory of Plant Genomics, Institute of Microbiology, Chinese Academy of Sciences, Beijing, 100101 China; 3 CAS Center for Excellence in Biotic Interactions, University of Chinese Academy of Sciences, Beijing, 100049 China

**Keywords:** Receptor-like cytoplasmic kinase, Biotic stress, Abiotic stress, Plant immunity

## Abstract

As sessile organisms, plants have to cope with environmental change and numerous biotic and abiotic stress. Upon perceiving environmental cues and stress signals using different types of receptors, plant cells initiate immediate and complicated signaling to regulate cellular processes and respond to stress. Receptor-like cytoplasmic kinases (RLCKs) transduce signals from receptors to cellular components and play roles in diverse biological processes. Recent studies have revealed the hubbing roles of RLCKs in plant responses to biotic stress. Emerging evidence indicates the important regulatory roles of RLCKs in plant responses to abiotic stress, growth, and development. As a pivot of cellular signaling, the activity and stability of RLCKs are dynamically and tightly controlled. Here, we summarize the current understanding of how RLCKs regulate plant responses to biotic and abiotic stress.

## Introduction

Both plant and animal cells can perceive internal signals, such as growth hormones or peptides, to regulate growth and developmental processes. Unlike animals, plants, as sessile organisms, have to cope with environmental change and stress. Plants have evolved a set of receptor proteins to sense and respond to internal and environmental signals. Among these, receptor-like kinases (RLKs) and receptor-like proteins (RLPs) are the major cell-surface receptors in terrestrial plants. Arabidopsis and rice genomes contain ~ 600 and ~ 1100 RLK members, respectively (Shiu et al., 2004). Plant RLKs possess a highly variable ectodomain (ECD), a transmembrane domain, and a cytoplasmic kinase domain. ECD is potentially involved in ligand perception and the cytoplasmic kinase domain is responsible for signal transduction (Shiu et al., 2004; Tang et al., 2017). There are ~ 170 and ~ 90 RLPs in Arabidopsis and rice, respectively, which are analogous to RLKs except for the lack of the intracellular kinase domain. Thus, RLPs relay signals through interactions with other signaling components. Plant RLKs and RLPs recognize a variety of ligands, including hormones, peptides and other signaling molecules from plant cells or the environment, thereby regulating plant growth, development, adaption to abiotic stress, and plant-microbe interactions (Couto and Zipfel, 2016; Tang et al., 2017; Yu et al., 2017). To date, a number of RLKs and RLPs, and their corresponding ligands have been identified. For instances, the RLK protein BRASSINOSTEROID INSENSITIVE 1 (BRI1) recognizes the steroid hormone brassinosteroid (BR) in Arabidopsis (Wang et al., 2001). Arabidopsis RLK protein FLAGELLIN SENSING 2 (FLS2) sense bacterial flagellin (or the epitope flg22) (Go’mez-Go’mez and Boller, 2000). Rice RLP CHITIN ELICITOR-BINDING PROTEIN (CEBiP1) recognizes fungal cell wall-derived chitin, Arabidopsis RLP RECEPTOR LIKE PROTEIN 23 (RLP23) recognizes necrosis and ethylene-inducing peptide 1-like proteins (NLPs) (Albert et al., 2015; Kaku et al., 2006). Therefore, these receptors with known ligands are referred to as receptor kinases (RKs) or receptor proteins (RPs) in this text.

**Fig. 1:**
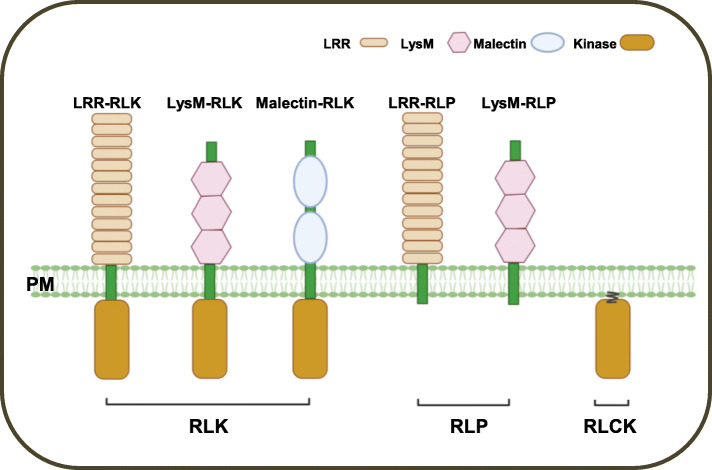
Domain organization of representative receptor-like kinases (RLKs), receptor-like proteins (RLPs), and receptor-like cytoplasmic kinases (RLCKs). Representative types of RLKs as well as RLPs harbor a transmembrane domain to get localized to the plasma membrane (PM). Many RLCKs associate with the plasma membrane (PM). LRR, leucine rich repeat; LysM, lysine motif

Plant RLKs and RLPs can be divided into many subfamilies based on the structurally difference of the ECD (Fig. 1), which contains the leucine-rich repeat (LRR) domain, lysin motifs (LysM), malectin, lectin, or epidermal growth factor-like (EGF) domain (Shiu et al., 2004; Vij et al., 2008). Intriguingly, one of the RLK subfamilies lacks the transmembrane domain and is referred to as a receptor-like cytoplasmic kinase (RLCK) subfamily. There are 149 members in Arabidopsis and 379 in rice, respectively (Shiu et al., 2004; Vij et al., 2008). Based on sequence homology, RLCKs can be divided into 17 subgroups. Most of them contain only a Ser/Thr kinase domain, analogous to RLKs (Liang and Zhou, 2018; Lin et al., 2013; Sun and Zhang, 2020).

Plant RLCKs have been reported to regulate a variety of biological processes including plant innate immunity, hormone signaling, sexual reproduction, stomatal patterning and adaptation to abiotic stress (Liang and Zhou, 2018; Lin et al., 2013; Sun and Zhang, 2020). Increasing evidence has revealed that plant RLCKs function in concert with RKs/RPs. Many RLCK members have been reported to be physically or genetically coupled to plant RKs or RPs to transduce receptor-mediated signaling (Tang et al., 2017; Yu et al., 2017). BOTRYTIS-INDUCED KINASE 1(BIK1), a typical representative member of Arabidopsis RLCK-VII, directly interacts with FLS2 and ELONGATION FACTOR-TU (EF-Tu) RECEPTOR (EFR) to regulate PAMP-activated signaling (Lu et al., 2010; Zhang et al., 2010). OsRLCK176 and OsRLCK185 interact with rice CHITIN ELICITOR RECEPTOR KINASE1 (OsCERK1) and are essential for chitin- and PGN-induced immune signaling (Ao et al., 2014; Yamaguchi et al., 2013). Arabidopsis MARIS (MRI), an RLCK-VIII member, has been reported to interact with ANXUR1 (ANX1), ANX2 and FERONIA (FER) to regulate integrity of pollen tube and growth of root hair (Boisson-Dernier et al., 2015). Arabidopsis COLD-RESPONSIVE PROTEIN KINASE1 (CRPK1) negatively regulates cold responses by phosphorylating 14-3-3 proteins (Liu et al., 2017). In this review, we focus on recent advances of RLCKs in regulating plant responses to biotic and abiotic stress.

## RLCKs are pivot signaling points in plant responses to biotic stress

**Fig. 2:**
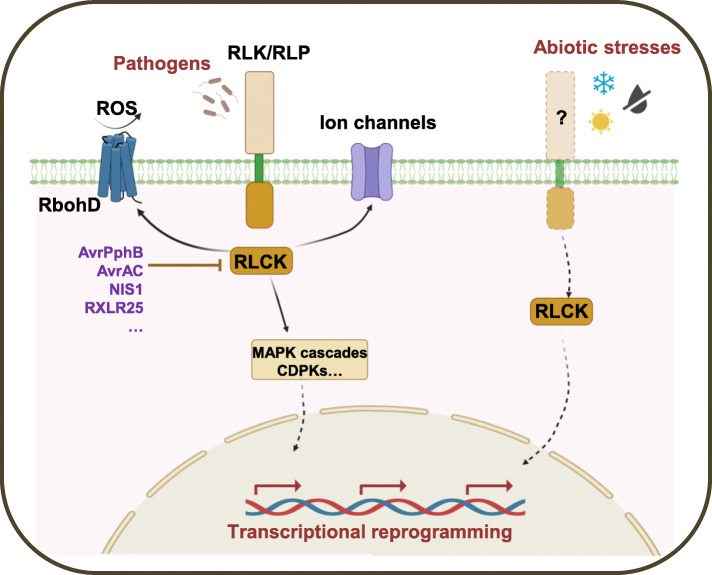
Signaling responses of RLCKs to biotic and abiotic stress in plants. RLCKs function downstream of RLK (or RLP) immune receptors to regulate a series of immune responses including production of reactive oxygen species (ROS), calcium influx, activation of MAPK cascades and CDPKs, and transcriptional reprogramming. As the central regulators of plant immunity, RLCKs are frequently targeted by microbial effectors such as bacterial effectors avrPphB and avrAC, the fungal effector NIS1, and the oomycete effector RXLR25. Increasing evidence indicates a role of RLCKs in orchestrating plant adaption to abiotic stress

Although plants have not evolved an adaptive immune system, they are equipped with a sophisticated innate immune system to prevent pathogen infection (Jones and Dangl, 2006; Chisholm, et al., 2006; Zhou and Zhang, 2020). RLCKs have been shown to regulate plant resistance to fungal and bacterial pathogens (Liang and Zhou, 2018; Lin et al., 2013; Sun and Zhang, 2020). Some RLCKs have also been shown to regulate plant resistance to viral pathogens and insects (Lee and Kim, 2015; Rashid et al., 2017; Sun et al., 2021), indicating a broad role of RLCKs in regulating plant responses to biotic stress (Fig. 2).

Upon immune activation, RLCKs integrate signaling from cell-surface-localized immune receptors. Plant RKs and RPs serve as immune receptors to perceive molecular patterns from microbes and danger signals from host cells caused by pathogen infection. These signaling molecules are known as microbe- or pathogen-associated molecular patterns (MAMPs/PAMPs) and damage-associated molecular patterns (DAMPs). The corresponding RKs/RPs are referred to as pattern recognition receptors (PRRs) (Couto and Zipfel, 2016; DeFalco and Zipfel, 2021; Tang et al., 2017; Yu et al., 2017). Arabidopsis FLS2 and EFR recognize bacterial flagellin and EF-Tu in the presence of the co-receptor BRI1-ASSOCIATED KINASE1 (BAK1) (Chinchilla et al., 2007; Heese et al., 2007). Arabidopsis CHITIN ELICITOR RECEPTOR KINASE1 (CERK1), LYSINE MOTIF RECEPTOR KINASE5 (LYK5), and rice CEBiP1 can recognize the fungal cell wall component chitin (Cao et al., 2014; Kaku et al., 2006; Liu et al., 2012; Miya et al., 2007; Wan et al., 2008). PEP RECEPTOR 1 (PEPR1) and PEPR2 can sense host-derived peptide peps (Krol et al., 2010). The recognition of MAMPs/PAMPs/DAMPs by PRRs trigger downstream PAMP-triggered immunity (PTI), which involves the production of reactive oxygen species (ROS), induction of calcium influx, activation of mitogen-activated protein kinase (MAPK) and calcium-dependent protein kinase (CDPK) pathways, and transcriptional reprogramming (DeFalco and Zipfel, 2021). Pathogenic microbes can evade host immune recognition or suppress host immunity by secreting effector proteins into hosts (Dangl et al., 2013; Dodds and Rathjen, 2010; Jones and Dangl, 2006). To counteract, plants have evolved intracellular immune receptors, which are known as nucleotide-binding and leucine-rich repeat domain-containing receptors (NLRs), to recognize cytoplasmic effectors (Jones et al., 2016). Plant cells have a limited number of NLRs to cope with numerous effector proteins, thus, plant NLR proteins recognize effectors through multiple strategies. The activation of NLRs leads to effector-triggered immunity (ETI), a more robust immune response, and is often accompanied by programmed cell death (Jones et al., 2016). Recent studies have shown that PTI and ETI share common signaling components and ETI functions through augment of PTI (Ngou et al., 2021; Pruitt et al., 2021; Tian et al., 2021; Yuan et al., 2021). RLCKs function as central kinases in the activation of plant immunity and are the major executors to activate and transduce immune signaling downstream of PRRs (DeFalco and Zipfel, 2021; Liang and Zhou, 2018). RLCKs are frequently attacked by microbial virulence effectors and are required for ETI activation (Liang and Zhou, 2018; Sun and Zhang, 2020).

### Phosphorylation-dependent regulation of ROS production

The activation of PRRs and NLRs triggers a series of immune responses. ROS production is an early immune response that is mainly controlled by plasma membrane-localized RESPIRATORY BURST OXIDASE HOMOLOG (Rboh) proteins (Qi et al., 2017). The apoplastic ROS functions as molecular signals to further regulate downstream immune responses, such as PAMP-induced stomatal closure and callose deposition to fend off the entry of phytopathogens (Castro et al., 2021; Daudi et al., 2012; Qi et al., 2017; Waszczak et al., 2018).

RbohD and RbohF are major proteins that control immune-related ROS production in Arabidopsis (Qi et al., 2017). RbohD has been shown to be regulated by diverse mechanisms (Dubiella et al., 2013; Kadota et al., 2015; Kadota et al., 2014; Li et al., 2014; Zhang et al., 2009). RbohD directly interacts with BIK1 and is phosphorylated by BIK1 and its closest homolog PBS1-LIKE 1 (PBL1). The phosphorylation deficient form of RbohD fails to restore flg22-induced ROS burst and stomatal immunity in *rbohD*, indicating that phosphorylation of RbohD by BIK1 and PBL1 is required for the activation of RbohD (Kadota et al., 2014; Li et al., 2014; Ranf et al., 2014). Similarly, another RLCK-VII member, RPM1-INDUCED PROTEIN KINASE (RIPK) directly phosphorylates RbohD and positively regulates ROS production triggered by multiple PAMPs (Li et al., 2021). In contrast to BIK1 and RIPK, Arabidopsis RLCK-VII members PBL13 and CONSTITUTIVE DIFFERENTIAL GROWTH1 (CDG1) negatively regulate flg22-induced ROS burst. PBL13 directly interacts with and phosphorylates RbohD to regulate the turnover of RbohD (Lee et al., 2020; Lin et al., 2015). CDG1 negatively regulates flg22 and chitin-induced ROS by promoting the degradation of FLS2 and CERK1 (Yang et al., 2021).

PATTERN-TRIGGERED IMMUNITY COMPROMISED RECEPTOR-LIKE CYTOPLASMIC KINASE1 (PCRK1), a homolog of BIK1, is also required for the flg22-induced ROS burst (Kong et al., 2016; Sreekanta et al., 2015). RLCK-XII members BR SIGNALING KINASE1 (BSK1), BSK5, BSK7 and BSK8 are required for flg22-induced maximum ROS production (Majhi et al., 2021; Shi et al., 2013; Yan et al., 2018). Whether PCRK1 and BSKs regulate flg22-induced ROS in a manner similar to BIK1 remains unclear. Rice OsRLCK57, OsRLCK107, OsRLCK118, and OsRLCK176 have been reported to directly associate with OsCREK1 and regulate chitin- and PGN-induced ROS production (Li et al., 2017). OSRLCK176 and OsRLCK118 have been reported to interact with and phosphorylate OsRbohB, indicating that rice Rboh proteins are similarly regulated by RLCKs as in Arabidopsis (Fan et al., 2018). BROAD-SPECTRUM RESISTANCE 1 (OsBSR1), also known as OsRLCK278, is required for chitin-induced production of ROS and defense-related gene expression (Kanda et al., 2017; Sugano et al., 2018). Silencing of PTO-INTERACTIN 1 (PTI1), a tomato RLCK-VIII member, leads to decreased flg22-induced ROS production and compromised resistance to *Pseudomonas syringae* infection with an unknown mechanism (Schwizer et al., 2017).

It is worth noting that RLCK proteins regulate immune-related ROS production with redundancy and specificity. Rao et al (2018) constructed nine high-order mutants of the RLCK-VII subfamily (*rlck-vii-1* ~ *rlck-vii-9*) based on the phylogenetic tree and systematically analyzed their roles in PAMP-induced immune responses. While RLCK-VII-5,  -7, and - 8 are widely required for ROS production induced by different PAMPs, RLCK-VII-4 is specifically required for chitin-induced ROS (Rao et al., 2018). Compared to other RLCK-VII members, PBL30, PBL31, and PBL32 mainly regulate ROS production mediated by RLP receptors (Pruitt et al., 2021). PBL34, PBL35, and PBL36 have been reported to be required for 3-OH-C10:0-induced immune responses including ROS production (Luo et al., 2020).

### Regulation of calcium channels and calcium-dependent signaling

Calcium is recognized as one of the most important second messengers and is involved in diverse signaling events in eukaryotes. Calcium is essential for both PRR- and NLR-mediated immune activation (Bi and Zhou, 2021; DeFalco and Zipfel, 2021). PAMPs trigger a rapid calcium influx, which is required for the activation of downstream immune signaling (Boudsocq and Sheen, 2013). Calcium binding to the EF hand of RbohD is essential for the activation of RbohD (Boudsocq and Sheen, 2013). Consistent with this, a calcium influx occurs within seconds upon PAMP treatment and even earlier than the ROS burst. The elevation of cytoplasmic calcium concentration activates CDPKs. CPK4, CPK5, CPK6, and CPK11 have been reported to be required for the activation of PAMP-induced immunity. The *cpk* multiple mutant showed reduced flg22-induced ROS production, defense-related gene expression, and bacterial resistance (Boudsocq et al., 2010). CPK5 has also been reported to directly phosphorylate RbohD to regulate ROS production (Dubiella et al., 2013).

BIK1 and PBL1 are required for flg22-induced calcium bursts. The *bik1 pbl1* mutant showed significantly reduced calcium bursts compared to that of wild-type plants (Li et al., 2014; Ranf et al., 2014). Consistent with this, co-expression of CPK5 with BIK1 K105E mutation, a dominant negative mutation of BIK1, blocks the flg22-induced phosphorylation of CPK5, indicating that other PBLs may also contribute to flg22-indued calcium influx and CDPK activation (Li et al., 2014).

Recently, CYCLIC NUCLEOTIDE GATED CHANNEL 2 (CNGC2) and CNGC4 have been reported to form a calcium channel that is responsible for flg22-induced calcium influx under high external calcium conditions. BIK1 directly phosphorylates CNGC2 and CNGC4 to activate calcium channels (Tian et al., 2019). Thor et al (2020) showed that BIK1 and PBL1 rapidly phosphorylate OSCA1.3, which activates its calcium channel activity in guard cells and promotes PAMP-induced stomatal closure (Thor et al., 2020). Similarly, OsRLCK185 was reported to phosphorylate OsCNGC9 to regulate chitin-induced calcium influx (Wang et al., 2019b). Another family of calcium channels, GLUTAMATE RECEPTOR 2.7 (GLR2.7), 2.8 and 2.9, has also been reported to contribute to PAMP-induced calcium influx (Bjornson et al., 2021). Whether GLR proteins are regulated by RLCKs remains unclear.

Calcium-permeable channel CNGC20 is phosphorylated by BIK1 and a gain-of-function *cngc20-4* mutant showed enhanced PTI responses and ETI hypersensitive cell death (Zhao et al., 2021). The *cngc20* null mutant was also identified in a screening for suppressor of *bak1 serk4* cell death phenotype and CNGC20 was phosphorylated by BAK1 (Yu et al., 2019). Both BIK1 and BAK1-mediated phosphorylation promoted the protein stability of CNGC20 (Yu et al., 2019; Zhao et al., 2021). CNGC20 has been reported to form heteromeric complexes with CNGC19 (Yu et al., 2019; Zhao et al., 2021). However, whether CNGC19 and CNGC20 contribute to calcium influx in plant immunity remains uncertain.

Previous studies have shown that activation of NLRs triggers a prolonged calcium influx, which is indispensable for RESISTANCE TO *Pseudomonas SYRINGAE* PV MACULICOLA1 (RPM1) and HOPZ-ACTIVATED RESISTANCE1 (ZAR1)-mediated HR responses (El Kasmi et al., 2017; Grant et al., 2000; Wang et al., 2019a). *Xanthomonas campestris* effector protein AvrAC was recognized by ZAR1 resistosome, which is composed of AvrAC, the RLCK-VII member PBL2, the RLCK-XII member RESISTANCE RELATED KINASE1 (RKS1) and the NLR protein ZAR1(Wang et al., 2019a; Wang et al., 2019c). Bi et al (2021) showed that the ZAR1 resistosome forms a calcium-permeable channel to trigger immunity and cell death (Bi et al., 2021).

### Regulation of MAPK cascades

The MAPK cascade is composed of MAPK kinase kinase (MAPKKK), MAPK kinase (MAPKK), and MAPK itself. The MAPK cascade is one of the most conserved signaling pathways in both mammalian and plant cells. Perception of PAMP molecules activates two conserved MAPK cascades, which lead to the phosphorylation of MPK3, MPK6, and MPK4 within minutes (Meng and Zhang, 2013). While MPK4 is activated by MEKK1 and MKK1/2, MPK3 and MPK6 are activated by MAPKKK3/5 and MKK4/5 (Asai et al., 2002; Bi et al., 2018; Gao et al., 2008; Qiu et al., 2008; Suarez-Rodriguez et al., 2007; Yan et al., 2018).

How PRRs transduce the signals to MAPKKKs had remained one of the key issues in the study of plant immunity. Plant RLCKs have been reported to be genetically required for PAMP-induced MAPK activation. AvrAc, a *Xanthomonas campestris* effector with uridylyl transferase activity, specifically suppresses the activation of BIK1 and its homologs (Feng et al., 2012). Expression of AvrAc in Arabidopsis significantly reduced the activation of MPK3/6 and MPK4 (Feng et al., 2012), indicating that RLCKs are required for the activation of MAPKs. Consistent with this, flg22-induced MAPK activation was reduced in *pcrk1* *pcrk2* double mutant (Kong et al., 2016). Pep2-induced MAPK activation was slightly reduced in the *bik1 pbl1* mutant (Yamada et al., 2016b). Rice OsRLCK185 and OsRLCK176 have been reported to interact with OsCERK1 and are required for chitin- and PGN-induced MAPK activation (Ao et al., 2014; Yamaguchi et al., 2013). PBL27, the Arabidopsis homolog of OsRLCK185, is required for chitin-triggered activation of MPK3/6. Rao et al (2018) systemically analyzed PAMP-induced MAPK activation in the nine *rlck-vii* high-order mutants and showed that the RLCK-VII-4 subgroup (PBL19, PBL20, PBL37, PBL38, PBL39 and PBL40) is specifically required for chitin-triggered MAPK activation (Rao et al., 2018). The RLCK-VII-8 subgroup (BIK1, PBL1, PBL11, PBL9 and PBL10) specifically regulate Pep2-induced MAPK activation (Rao et al., 2018).

Over the last few years, reports have shown that RLCKs directly phosphorylate MAPKKKs to regulate PAMP-induced MAPK activation. Arabidopsis PBL27 interacts with both CERK1 and MAPKKK5 and phosphorylates MAPKKK5 to modulate chitin-induced MAPK (Yamada et al., 2016a). BSK1 directly phosphorylates MAPKKK5 and regulates plant resistance (Yan et al., 2018). Later studies showed that OsRLCK185 interacts with OsCERK1 and OsMAPKKKε and functions in plant resistance to rice blast (Wang et al., 2017). Bi et al (2018) showed that the RLCK-VII subfamily directly links PRR and MAPKKKs, and RLCK-VII-4 members phosphorylate MAPKKK5 to activate chitin-induced MAPK (Bi et al., 2018; Rao et al., 2018).

### Regulatory roles of RLCKs in NLR-mediated signaling

Pathogenic microbes are equipped with a variety of virulence effectors that target key signaling components in plant immunity. As the central kinases in immune signaling, RLCKs are frequently targeted by microbial effector proteins to suppress host immunity (Liang and Zhou, 2018; Sun and Zhang, 2020). Some of the RLCKs work as sensors for intracellular effectors and they are regarded as “guardees” or “decoys” to perceive the presence of effectors (Liang and Zhou, 2018; Sun and Zhang, 2020). Tomato Pto protein is the first characterized effector-targeted RLCK in plants. Pto directly interacts with *Pseudomonas syringae* effectors AvrPto and AvrPtoB and activates ETI through the NLR protein PTO RESISTANCE AND FENTHION SENSITIVITY (Prf) (Kim et al., 2002; Scofield et al., 1996; Tang et al., 1996). AvrPto and AvrPtoB were also reported to target the kinase domain of FLS2 and BAK1 to suppress plant immune responses (Shan et al., 2008; Xiang et al., 2008). Thus, Pto serves as a decoy to protect FLS2 -BAK1 complex and functions as a sensor to perceive avrPto and avrPtoB.

*Pseudomonas syringae* effector AvrPphB is a cysteine protease that proteolytically cleaves AVRPPHB SUSCEPTIBLE 1 (PBS1), an RLCK-VII member, and leads to the activation of effector-triggered immune response through the NLR protein RESISTANCE TO *Pseudomonas Syringae*5 (RPS5) (Ade et al., 2007). PRS5 monitors the cleavage of PBS1 to detect the presence of AvrPphB (Ade et al., 2007; Shao et al., 2003). Later research revealed that AvrPphB also cleaves other close homologs of PBS1, the PBS1-like (PBL) proteins, to suppress plant immune responses. This led to the identification of BIK1 and other PBL proteins as central components of immune signaling (Zhang et al., 2010).

*Xanthomonas campestris* effector AvrAC (also known as XopAC) promotes bacterial virulence by uridylylating the conserved phosphorylation sites in BIK1 and related PBLs to suppress plant immunity (Feng et al., 2012). The NLR protein ZAR1 forms a stable complex with RESISTANCE RELATED KINASE1 (RKS1), a pseudokinase from the RLCK-XII subgroup. The recognition of AvrAc by ZAR1 also requires the RLCK-VII member PBL2 (Guy et al., 2013; Wang et al., 2015). Once PBL2 is uridylylated by AvrAc, the RKS1-ZAR1 complex recruits PBL2 and induces the oligomerization of ZAR1 to form a resistosome, which serves as a calcium channel to trigger immunity and cell death (Bi et al., 2021; Wang et al., 2015; Wang et al., 2019a; Wang et al., 2019c).

HopZ1a is a *Pseudomonas syringae* effector with acetyltransferase activity that also triggers ETI through the NLR protein ZAR1. ZAR1 recognizes the presence of HopZ1a through HOPZ-ETI-DEFICIENT1 (ZED1), a pseudokinase from the RLCK-XII subgroup. ZED1 interacts with both ZAR1 and HopZ1a and serves as a decoy substrate for HopZ1a recognition (Lewis et al., 2013). Two closely related RLCK-VII members, SUPPRESSOR OF ZED1-D1 (SZE1) and SZE2, have been reported to interact with ZED1 and ZAR1 and are involved in the recognition of HopZ1a (Liu et al., 2019). Likewise, ZAR1 also recognizes the virulence effector HopF2a through ZRK3, a homolog of ZED1 (Seto et al., 2017).

OsRLCK185 and OsRLCK55 directly interact with *Xanthomonas oryzae* effector Xoo1488 in yeast two-hybrid assays (Yamaguchi et al., 2013). Further studies showed that OsRLCK185 interacts with OsCERK1 to regulate chitin-induced immune signaling (Yamaguchi et al., 2013). Consistent with this, Xoo1488 suppresses chitin-induced immune responses, indicating that OsRLCK185 is a virulence target of Xoo1488. The oomycete pathogen *Phytopthora capsici* effector RXLR25 and *Colletotrichum* fungi effector NIS1 have been reported to suppress PAMP-induced phosphorylation of RLCK-VII proteins by direct interaction (Irieda et al., 2019; Liang et al., 2021).

A tomato RLCK named AVR9/CF-9 INDUCED KINASE 1 (ACIK1) is required for Avr9/Cf9 and Avr4/Cf4-mediated resistance (Rowland et al., 2005). The Arabidopsis NLR protein RPM1 detects *Pseudomonas syringae* effector AvrB and AvrPRM1 by monitoring RPM1-INTERACTING 4 (RIN4) phosphorylation. Although neither effector has kinase activity, it causes RIN4 phosphorylation through the host RLCK protein RIPK and CDG1 with an unknown mechanism (Chung et al., 2011; Liu et al., 2011; Yang et al., 2021). The *Pseudomonas syringae* effector HopAI1 directly targets MPK3, MPK4 and MPK6 and inactivates MAPK cascades (Zhang et al., 2007). The disruption of the MAPK cascade is sensed by the NLR protein SUPPRESSOR OF MKK1 MKK2, 2 (SUMM2), which triggers cell death (Zhang et al., 2012). Zhang et al (2017) revealed that CALMODULIN-BINDING RECEPTOR-LIKE CYTOPLASMIC KINASE 3 (CRCK3) associates with SUMM2 and is directly phosphorylated by MPK4. SUMM2 monitors CRCK3 phosphorylation to sense the disruption of MAPK cascades (Zhang et al., 2017). A recent study showed that CRCK3 overexpression also activates cell death, which requires the kinase activity of CRCK3 and the NLR protein SUMM2 (Yang et al., 2020; Zhang et al., 2017).

## Emerging roles of RLCKs in plant responses to abiotic stress

In addition to pathogen infection, plants also have to cope with several abiotic stress including drought, salinity and low temperature (Zhu, 2016). Although it has not been extensively studied as in plant-microbe interactions, increasing studies have reported that RLCK proteins play important regulatory roles in plant responses to many abiotic stresses. In the case of rice, for instance, 86 of the 376 rice *RLCK* genes were differentially expressed in response to cold, salt, and dehydration stimuli (Vij et al., 2008). OsRLCK311 has been reported to play a positive role in salt tolerance (Sade et al., 2020; Zhang et al., 2021). *OsRLCK241* was transcriptionally induced by salt and drought. OsRLCK241 overexpression confers enhanced resistance to salt and drought stress (Zhang et al., 2021).

Arabidopsis ABA- AND OSMOTIC STRESS-INDUCIBLE RECEPTOR-LIKE CYTOPLASMIC KINASE1 (ARCK1) has been reported to interact with CYSTEINE-RICH RLK 36 (CRK36), a cysteine-rich repeat RLK, to regulate tolerance to osmotic stress and ABA. The *arck1* mutant displayed reduced tolerance to osmotic and ABA stress. Further studies showed that CRK36 directly phosphorylates ARCK1 and regulates the expression of stress-responsive genes via an unknown mechanism (Tanaka et al., 2012). Arabidopsis CRCK1 kinase has been reported to be induced under multiple stress conditions, including cold, salt, and ABA. Whether and how CRCK1 is involved in abiotic stress tolerance remains unclear (Yang et al., 2004). Esi47 is a homolog of Arabidopsis PCRK1 in the wheatgrass and is upregulated by salt stress and ABA treatment (Shen et al., 2001). The Arabidopsis RLCK-VIII member CYTOSOLIC ABA RECEPTOR KINASE 1 (CARK1) and CARK6 directly interact with and phosphorylate subfamily III ABA receptors, and positively regulate drought stress (Li et al., 2019; Wang et al., 2019d; Zhang et al., 2018a). The rice RLCK protein, SALT TOLERANCE RECEPTOR-LIKE CYTOPLASMIC KINASE 1 (STRK1), positively regulates salt and oxidative stress by interacting with CatC at the plasma membrane. STRK1 has been reported to phosphorylate CatC at specific sites to enhance the catalase activity of CatC (Zhou et al., 2018).

The soybean RLCK GsRLCK, from wild soybean *Glycine soja*, is upregulated by salt, alkali, drought, and ABA. GsRLCK overexpression leads to increased tolerance to drought and salt stress in Arabidopsis (Sun et al., 2013). CALCIUM-DEPENDENT CALMODULIN-BINDING RECEPTOR-LIKE KINASE (GsCBRLK), an RLCK-VI member from *Glycine soja*, is transcriptionally induced by salt, drought, cold and ABA. GsCBRLK interacts with a group 3 late embryogenesis abundant protein GsPM30, and overexpression of GsCBRLK in Arabidopsis has greatly enhanced plant tolerance to salt and ABA (Sun et al., 2019; Yang et al., 2010). GsCBRLK also interacts with a methionine sulfoxide reductase (MSR) B protein GsMSRB5a and activates ROS signaling to regulate carbonate alkaline stress (Sun et al., 2016).

Rice OsRLCK253 was identified in a search for rice SAP1-interacting proteins. SAP1 is known to confer tolerance to abiotic stress. OsRLCK253 associates with SAP1 and SAP11. OsRLCK253 overexpression causes increased resistance to salt and drought stress in Arabidopsis (Giri et al., 2011). Rice GROWTH UNDER DROUGHT KINASE (GUDK) has been reported to be required for grain yield under drought condition. The *gudk* mutant displays defects in response to salt stress, osmotic stress, and ABA treatment. GUDK phosphorylates a transcription factor, OsAP37, involved in drought tolerance (Ramegowda et al., 2015; Ramegowda et al., 2014).

Liu et al (2017) reported that Arabidopsis CRPK1 negatively regulates cold tolerance by phosphorylating 14-3-3 proteins upon cold treatment. COLD-RESPONSIVE C-REPEAT-BINDING FACTORs (CBFs) are key transcription factors that promote cold tolerance. The phosphorylated 14-3-3 proteins then translocate into the nucleus and destabilize CBFs to regulate cold responses (Liu et al., 2017). Together, how RLCKs regulate abiotic stress responses requires further elucidation. Additional RLCKs functioning in plant responses to abiotic stress remain to be discovered.

## Multi-layered regulation on the activity and stability of RLCKs

As mentioned above, RLCKs are the central kinases in plant immune signaling (Couto and Zipfel, 2016; DeFalco and Zipfel, 2021; Liang and Zhou, 2018; Sun and Zhang, 2020) and important regulators in plant responses to abiotic stress (Liang and Zhou, 2018; Lin et al., 2013). Thus, the activity and stability of RLCKs must be tightly regulated to ensure appropriate responses to biotic and abiotic stress (Liang and Zhou, 2018; Sun and Zhang, 2020).

BIK1 is a representative member of plant RLCK and has been extensively studied over the last decade. Upon flg22 perception, FLS2 forms a complex with BAK1 and leads to the phosphorylation of BIK1 at specific sites, which causes the dissociation of BIK1 from the FLS2 receptor complex (Lu et al., 2010; Zhang et al., 2010). Activated BIK1 phosphorylates its downstream targets to transduce immune signals (Kadota et al., 2014; Li et al., 2014; Liang et al., 2016, 2018). BIK1 is also regulated by monoubiquitination, which is caused by a pair of E3 ligases, RHA3A and RHA3B. The monoubiquitination of BIK1 is required for the flg22-induced BIK1-FLS2 dissociation and the full function of BIK1 (Ma et al., 2020).

To ensure that the immune responses are controlled at appropriate amplitude, the activity and stability of BIK1 are tightly controlled. The Arabidopsis protein phosphatase PP2C38 interacts with BIK1, and controls the phosphorylation status of BIK1 (Couto et al., 2016). PP2C38 dephosphorylates BIK1 in the resting state, and upon PAMP treatment, it gets phosphorylated and dissociates from BIK1 to ensure BIK1 activation (Couto et al., 2016). Similarly, a PP2C phosphatase, Pic1, negatively regulates the phosphorylation status of the tomato RLCK protein Pti1b to modulate immune activation (Giska and Martin, 2019).

BIK1 is known to be degraded through the ubiquitin-proteasome pathway, and its stability has been reported to be differently regulated by CPK28, a calcium-dependent protein kinase (Monaghan et al., 2014), and heterotrimeric G protein complex composed of EXTRA-LARGE G PROTEIN 2/3 (XLG2/3), AGB1 and AGG1/2 (Liang et al., 2016). CPK28 interacts with BIK1 and negatively regulates BIK1 accumulation, as the *cpk28* mutant shows increased BIK1 protein accumulation and enhanced disease resistance (Monaghan et al., 2014). In contrast, Arabidopsis G proteins positively regulate BIK1 accumulation. The *xlg2 xlg3*, *agb1* and *agg1 agg2* mutant plants showed reduced BIK1 protein levels and compromised immune responses (Liang et al., 2016). PBL20, another RLCK-VII member, showed enhanced degradation in G protein mutant extracts, indicating that the G protein also regulates the stability of other RLCKs (Liang et al., 2016). Wang et al (2018) identified a pair of E3 ligases, PLANT U-BOX25 (PUB25) and PUB26, which are responsible for the proteasome-mediated degradation of BIK1 (Wang et al., 2018). While G proteins negatively regulate the E3 ligase activity of PUB25/26, CPK28 positively regulates the activity of PUB25/26 by phosphorylation (Wang et al., 2018). Together, these reports showed that CPK28 and G proteins coordinate the turnover of BIK1 by controlling the E3 ligase activity of PUB25/26. In addition, two MAP4Ks, SIK1 and MAP4K4, have been reported to directly phosphorylate BIK1 and positively regulate BIK1 stability (Jiang et al., 2019; Zhang et al., 2018b). The E3 ligase PUB4 has been reported to promote BIK1 degradation before immune activation. After PAMP perception, PUB4 positively regulates the accumulation of activated BIK1 (Derkacheva et al., 2020). Taken together, these reports showed that the stability of BIK1 is tightly controlled by multiple layered regulations.

## Conclusions and perspectives

Recent advances have documented the key regulatory roles of RLCKs in plant responses to biotic stress. Plant RLCKs directly associate with RK or RP immune receptors to regulate PRR-mediated signaling. RLCKs target diverse substrates to transduce immune signaling via phosphorylation. To date, MAPKKKs, NADPH oxidase, calcium channels, and G proteins have been identified as substrates for RLCKs (DeFalco and Zipfel, 2021; Zhou and Zhang, 2020). On the other side, modifications of RLCKs trigger the activation of NLR-mediated immune signaling. Most recent studies also implied the contribution of RLCKs in connecting PRR- and NLR-mediated signaling (Ngou et al., 2021; Pruitt et al., 2021; Tian et al., 2021; Yuan et al., 2021). Additional types of substrates and modes of RLCK actions in NLR-mediated signaling remain to be identified in the future.

Increasing evidence also indicates the important roles of RLCKs in plant responses to abiotic stress. However, the underlying mechanisms governing the activation of RLCKs remain less investigated. Several RLKs have been proved to function in plant responses to abiotic stress. Whether RLK/RLP-mediated RLCK phosphorylation is a common mechanism governing RLCK activation remains elusive.

Futhermore, the mechanisms governing RLCK signaling specificity require further investigation. Previous studies have revealed the functional specificities of a subset of RLCK-VII subfamily members in different PRR-mediated signaling. In addition, BIK1 was shown to play opposite roles in different PRR-mediated signaling pathways (Wan et al., 2019). The evidence indicates the functional specificities of RLCKs in signaling processes. Future studies are required to further determine the specificity and regulatory mechanisms of signaling integration and dispersal by RLCKs in regulating plant responses to biotic and abiotic stress, growth and development.

## Data Availability

Not applicable.

## Abbreviations

RLCK: Receptor-like cytoplasmic kinase
RLK: Receptor-like kinase
RLP: Receptor-like protein
RK: Receptor kinase
RP: Receptor protein
ECD: Ectodomain
ANX1: ANXUR 1
BR: Brassinosteroid
BRI1: Brassinosteroid insensitive 1
BAK1: BRI1-associated receptor kinase 1
FLS2: Flagellin sensing 2
CEBiP1: Chitin elicitor-binding protein 1
RLP23: Receptor like protein 23
NLP: Necrosis and ethylene-inducing peptide 1-like protein
LRR: Leucine-rich repeat
LysM: Lysine motif
EGF: Epidermal growth factor
BIK1: Botrytis-induced kinase 1
EFR: Elongation factor-tu receptor
MRI: MARIS
FER: FERONIA
CRPK1: Cold-responsive protein kinase 1
PAMP/MAMP: Pathogen/microbe-associated molecular pattern
CDPK: Calcium-dependent protein kinase
DAMP: Damage-associated molecular pattern
LYK5: Lysine motif receptor kinase 5
ETI: Effector-triggered immunity
EF-Tu: Elongation factor Tu
MAPK: Mitogen-activated protein kinase
PEP1: Plant elicitor peptide 1
PEPR1: Pep receptor 1
PRR: Pattern recognition receptor
PTI: Pamp-triggered immunity
NLR: Nucleotide-binding and leucine-rich repeat domain-containing receptor
ROS: Reactive oxygen species
Rboh: Respiratory burst oxidase homolog
SUMM2: Suppressor of MKK1 MKK2 2
PBL1: PBS1-like 1
RIPK: RPM1-induced protein kinase
CDG1: Constitutive differential growth 1
PCRK1: Pattern-triggered immunity compromised receptor-like cytoplasmic kinase 1
BSK1: BR signaling kinase 1
BSR1: Broad-spectrum resistance 1
PTI1: PTO-interactin 1
CNGC: Cyclic nucleotide gated channel
GLR2.7: Glutamate receptor
RPM1: Resistance to *Pseudomonas SYRINGAE* PV maculicola 1
ZAR1: Hopz-activated resistance 1
RKS1: Resistance related kinase 1
Prf: PTO resistance and fenthion sensitivity
PBS1: AVRPPHB susceptible 1
RPS5: Resistance to *Pseudomonas SYRINGAE* 5
RKS1: Resistance related kinase 1
ZED1: Hopz-Eti-deficient 1
SZE1: Suppressor of Zed1-D1
ACIK1: AVR9/CF-9 induced kinase 1
RIN4: RPM1-interacting 4
CRCK3: Calmodulin-binding receptor-like cytoplasmic kinase 3
ARCK1: ABA- and osmotic stress-inducible receptor-like cytoplasmic kinase 1
CRK36: Cysteine-rich RLK 36
CARK1: Cytosolic aba receptor kinase 1
STRK1: Salt tolerance receptor-like cytoplasmic kinase 1
GsCBRLK: Calcium-dependent calmodulin-binding receptor-like kinase
GUDK: Growth under drought kinase
CBF: Cold-responsive C-repeat-binding factor
XLG: Extra-large G protein
PUB25: Plant U-Box 25
